# Association of day-of-injury plasma glial fibrillary acidic protein concentration and six-month posttraumatic stress disorder in patients with mild traumatic brain injury

**DOI:** 10.1038/s41386-022-01359-5

**Published:** 2022-06-18

**Authors:** Jacqueline R. Kulbe, Sonia Jain, Lindsay D. Nelson, Frederick K. Korley, Pratik Mukherjee, Xiaoying Sun, David O. Okonkwo, Joseph T. Giacino, Mary J. Vassar, Claudia S. Robertson, Michael A. McCrea, Kevin K. W. Wang, Nancy Temkin, Christine L. Mac Donald, Sabrina R. Taylor, Adam R. Ferguson, Amy J. Markowitz, Ramon Diaz-Arrastia, Geoffrey T. Manley, Murray B. Stein, TRACK-TBI Investigators, Neeraj Badjatia, Ann-Christine Duhaime, V. Ramana Feeser, C. Dirk Keene, Christopher Madden, Randall Merchant, Ava Puccio, David Schnyer, Sabrina R. Taylor, Alex Valadka, John K. Yue, Esther L. Yuh, Ross Zafonte

**Affiliations:** 1 Department of Psychiatry, University of California, San Diego, La Jolla, CA USA; 2 Biostatistics Research Center, Herbert Wertheim School of Public Health and Human Longevity Science, University of California, San Diego, La Jolla, CA USA; 3 Departments of Neurosurgery and Neurology, Medical College of Wisconsin, Milwaukee, WI USA; 4 Department of Emergency Medicine, University of Michigan, Ann Arbor, MI USA; 5 Department of Radiology & Biomedical Imaging, UCSF, San Francisco, CA USA; 6 Department of Bioengineering & Therapeutic Sciences, UCSF, San Francisco, CA USA; 7 Department of Neurological Surgery, University of Pittsburgh Medical Center, Pittsburgh, PA USA; 8 Department of Physical Medicine and Rehabilitation, Harvard Medical School, Boston, MA USA; 9 Spaulding Rehabilitation Hospital, Charlestown, MA USA; 10 Brain and Spinal Cord Injury Center, Zuckerberg San Francisco General Hospital and Trauma Center, San Francisco, CA USA; 11 Department of Neurological Surgery, UCSF, San Francisco, CA USA; 12 Department of Neurosurgery, Baylor College of Medicine, Houston, TX USA; 13 Department of Emergency Medicine, University of Florida, Gainesville, FL USA; 14 Department of Neurological Surgery, University of Washington, Seattle, WA USA; 15 Department of Neurology, University of Pennsylvania, Philadelphia, PA USA; 16 School of Public Health, University of California, San Diego, La Jolla, CA USA; 17 VA San Diego Healthcare System, San Diego, CA USA; 18 University of Maryland, College Park, USA; 19 MassGeneral Hospital for Children, Boston, USA; 20 Virginia Commonwealth University, Richmond, USA; 21 University of Washington, Seattle, USA; 22 UT Southwestern, Dallas, USA; 23 UT Austin, Austin, USA; 24 University of California, San Francisco, USA

**Keywords:** Diagnostic markers, Trauma

## Abstract

Several proteins have proven useful as blood-based biomarkers to assist in evaluation and management of traumatic brain injury (TBI). The objective of this study was to determine whether two day-of-injury blood-based biomarkers are predictive of posttraumatic stress disorder (PTSD). We used data from 1143 individuals with mild TBI (mTBI; defined as admission Glasgow Coma Scale [GCS] score 13–15) enrolled in TRACK-TBI, a prospective longitudinal study of level 1 trauma center patients. Plasma glial fibrillary acidic protein (GFAP) and serum high sensitivity C-reactive protein (hsCRP) were measured from blood collected within 24 h of injury. Two hundred and twenty-seven (19.9% of) patients had probable PTSD (PCL-5 score ≥ 33) at 6 months post-injury. GFAP levels were positively associated (Spearman’s rho = 0.35, *p* < 0.001) with duration of posttraumatic amnesia (PTA). There was an inverse association between PTSD and (log)GFAP (adjusted OR = 0.85, 95% CI 0.77–0.95 per log unit increase) levels, but no significant association with (log)hsCRP (adjusted OR = 1.11, 95% CI 0.98–1.25 per log unit increase) levels. Elevated day-of-injury plasma GFAP, a biomarker of glial reactivity, is associated with reduced risk of PTSD after mTBI. This finding merits replication and additional studies to determine a possible neurocognitive basis for this relationship.

## Introduction

Many patients with mild TBI (mTBI; i.e., those with initial Glasgow Coma Scores [GCS] of 13–15) do not fully recover from their injury [1] and psychological health problems such as posttraumatic stress disorder (PTSD) frequently contribute to residual dysfunction and reduced quality of life [2, 3]. PTSD is seen in upwards of 20% of patients with mTBI and more commonly than in patients with non-head orthopedic injuries [4, 5].

The observation that PTSD is more common in association with head than non-head injuries has led to hypotheses that brain injury – likely involving damage to specific regions or disruption of connections to the hippocampus, frontal and cingulate cortex, insula or amygdala – explains, at least in part, this association [3, 5–7]. We have shown that smaller volume of several of these structures assessed 2 weeks post-injury, but still presumably reflecting the *pre-injury* state, are associated with greater likelihood of PTSD 3 months post-injury [8]. To the best of our knowledge, there are no studies that prospectively and systematically relate the extent of parenchymal injury to PTSD in patients with TBI. The absence of such data reflects the difficulty in obtaining standardized imaging measures post-injury, a problem that might be addressed by the availability of blood-based biomarkers of brain injury [9, 10].

Over the past several years there have been meaningful advances in the development and validation of blood-based biomarkers of traumatic brain injury [10]. In a prospective cohort study of 584 adult trauma patients seen at level I trauma centers, glial fibrillary acidic protein (GFAP) – which is believed to be a specific marker of astrocyte activation [11] – performed consistently well in detecting mild-to-moderate TBI and presence or absence of CT abnormalities [12]. A study of US military cadets during combat training showed that plasma levels of GFAP differentiated those with and without acute concussions at the acute post-injury point (<6 h) and at the 24–48 h post-injury point [13]. GFAP levels are elevated in athletes with sports-related concussion, and those with loss of consciousness (LOC) or posttraumatic amnesia (PTA) have significantly higher levels than athletes with concussion but neither LOC or PTA [14]. It has also recently been shown that day-of-injury plasma GFAP levels may be useful in the detection of brain injury on MRI even among patients with normal head CT scans [15]. Diagnostic aids for TBI based on GFAP and another blood-based biomarker– ubiquitin C-terminal hydrolase-L1 (UCH-L1) – have recently obtained regulatory approval (Banyan Brain Trauma Indicator Test. Banyan Biomarkers; 2018; i-STAT TBI Plasma Cartridge. Abbott Point of Care Inc. Abbott Park, IL; 2020) [16].

Whereas the aforementioned studies suggest that GFAP appears to be a useful blood-based biomarker of both the presence and initial severity of TBI, limited work has investigated its value in predicting longer-term outcomes such as the psychological conditions that are prevalent in the mTBI population. Here, using prospective longitudinal data from the multicenter Transforming Research and Clinical Knowledge in TBI (TRACK-TBI) study, we characterized the relationship between day-of-injury plasma GFAP, injury characteristics (in particular, PTA, considered to be a marker of injury severity), and 6-month probable PTSD. Our primary hypothesis was that increased GFAP levels would be associated with increased risk of PTSD post-injury. We chose to evaluate GFAP because its peak in plasma (approximately 20 h after injury with a slow decline thereafter through 72 h) is closest to the timing of the initial post-injury blood sample drawn in TRACK-TBI (within 24 h of injury) and is therefore likely to be a better indicator of the extent of parenchymal injury than other biomarker candidates such as UCH-L1, which rises rapidly and peaks at 8 h post-injury [12]. We further hypothesized that plasma GFAP within 24 h of injury would be associated with extent of posttraumatic amnesia, thereby reflecting another correlate of injury severity (though all within the initial GCS 13–15 range of mTBI).

Lastly, we compared and contrasted results with a blood-based biomarker of tissue injury and inflammation that is not brain-specific but has been shown to be predictive of 6-month disability after TBI [17], high sensitivity C-reactive protein (hsCRP). Noting that some studies have found an association between serum levels of hsCRP and PTSD symptoms [18], we also hypothesized that hsCRP levels would be associated with PTSD, but not as strongly as GFAP levels. In testing these hypotheses, we thought it important to take into consideration possibly confounding factors known to influence risk for PTSD in the context of mTBI (e.g., female sex, cause of injury, history of pre-injury mental disorder, and abnormalities on CT) [5, 19].

## Participants and methods

### Overview

TRACK-TBI is an 18-center prospective observational study of subjects evaluated in level I trauma centers within 24 h of injury from 2/26/2014 through 8/08/2018 [1]. We analyzed data from 1625 subjects, age > = 17 years, with GCS ED arrival score between 13 and 15, who were enrolled between March 2014 and July 2018, and had day-of-injury plasma GFAP and serum hsCRP measurements. Of these, 1143 completed the 6-month assessment. Inclusion criteria comprised having one’s treating physician order a head computed tomography (CT) scan due to suspicion of TBI; meeting the American Congress of Rehabilitation Medicine’s (ACRM) definition of TBI; enrollment for the study blood draw within 24 h of injury; adequate visual acuity/hearing pre-injury; and fluency in English or Spanish. Exclusion criteria included: significant polytrauma that would interfere with follow-up; penetrating TBI; prisoners or patients in custody; pregnancy; patients under psychiatric care without consent; major debilitating mental (e.g., schizophrenia or bipolar disorder) or neurological disorders (eg, stroke, dementia) or any other disorder that would interfere with assessment and follow-up or provision of informed consent; current participant in an interventional trial. Written informed consent was obtained from subjects or legally authorized representatives. The study was approved by the institutional review boards of enrolling sites.

Demographic and clinical data were obtained by experienced research assistants through medical record review and/or subject interviews. Head CT scans were sent to a central imaging repository and were read by a single board-certified neuroradiologist based on the Common Data Elements (CDE) in Radiologic Imaging of Traumatic Brain Injury [20].

### Measures

#### Primary outcome

PTSD Checklist for DSM-5 (PCL-5): The PCL-5 is a widely used measure of posttraumatic stress disorder symptoms. The range of the scale is 0–80. Signal detection analyses against a clinical gold standard revealed that PCL-5 cut scores of 31 to 33 were optimally efficient for diagnosing PTSD [21]. Consistent with our prior work in this area, we used scores of ≥ 33 to indicate probable PTSD [5].

#### Glasgow Coma Scale (GCS)

The GCS is a widely used estimate of brain injury severity that characterizes gross level of consciousness soon after injury (range 3–15; 13–15 is customarily considered “mild” TBI) [22].

#### Injury characteristics

The TRACK-TBI assessment obtained from the respondent and the medical record information about the characteristics of the injury included (a) cause of injury (e.g., motor vehicle collision; fall; other accidental injury; assault); (b) occurrence and duration of disturbance in consciousness or LOC; and (c) occurrence and duration of PTA.

#### Past medical history

The TRACK-TBI Interview requested information from the respondent (acquired at baseline, and in some cases collected from a relative or other suitable informant) about prior TBI(s) and prior history of mental disorder.

#### Blood sampling

Blood samples were obtained within 24 h of injury, processed, aliquoted, and stored in a −80 °C freezer within 2 h of collection. Sample acquisition, processing, and storage were performed following the TBI-CDEs Biospecimens and Biomarkers Working Group Guidelines [23]. Coded samples were then shipped overnight and on dry ice to a central repository, and from the central repository to the laboratory for analysis. Sample analysis occurred in a single laboratory (Abbott Laboratories, Abbott Park, IL) by personnel blinded to sample information. Plasma samples used in this analysis underwent one freeze-thaw cycle.

### Assays

Blinded sample analysis of hsCRP was carried out by a single laboratory (University College of Dublin) using the Abbott Architect c8000, MULTIGENT CRP Vario assay using the high-sensitivity method (CRP16). Anti-CRP antibodies adsorbed to latex particles agglutinate when an antigen-antibody reaction occurs with CRP, resulting in a change in absorbance proportional to the quantity of CRP in the sample. Serum samples were thawed in batches at room temperature and centrifuged at 1500 rfc for 10 min at 4 °C before testing. Assays were performed in duplicate with a lower limit of quantification of 0.1 mg/L and a reportable range of 0.1–160.0 mg/L.

The first batch of GFAP concentrations (*n* = 990) was measured using the prototype point-of-care i-STAT™ Alinity™ System. The second batch of GFAP concentrations (*n* = 635) was measured on the prototype core lab Abbott ARCHITECT^®^ platform for faster throughput. The i-STAT™ Alinity™ GFAP test uses the sandwich enzyme-linked immunosorbent assay (ELISA) method with electrochemical detection of the resulting enzyme signal. The GFAP assay’s reportable range was from 0 to 50,000 pg/mL. The limit of detection (LoD) and limit of quantitation (LoQ) were <15 pg/mL and <25 pg/mL, respectively. Within-laboratory precision, measured by the coefficient of variation (CV), was 2.8 to 14.2%.

The prototype ARCHITECT^®^ GFAP assay is a two-step sandwich assay that use a chemiluminescent microparticle immunoassay (CMIA) technology. The prototype GFAP assay calibration range was from 0 to 50,000 pg/mL. The LoD and LoQ were 2 pg/mL and 5 pg/mL, respectively, for a reportable range of 2–50,000 pg/mL. The within-laboratory CV was 2.0 to 5.6%. Samples with values greater than 50,000 pg/mL were retested with a 10-fold automated dilution protocol. All samples were tested neat, without dilution, and in duplicate. Samples reading greater than the calibration range were reported as greater than the reportable range and were not diluted. ARCHITECT^®^ GFAP values were converted to iSTAT equivalents using a previously derived and validated (Spearman’s correlation coefficient = 0.985) equation: iSTAT = −12.36 + 1.02 *ARCHITECT [24]. Technicians performing biomarker measurements were blinded to clinical outcome data.

### Statistical analysis

Demographics and clinical characteristics were summarized for the study cohort. Group comparisons used the Wilcoxon Rank Sum test for the continuous variables and Fisher’s exact test for categorical variables. Biomarker concentrations were not normally distributed and were summarized by reporting medians and their corresponding interquartile range. Log-transformed biomarker levels were used for modeling. Biomarker concentrations that were below the LoD were analyzed using the reported value and values above the assay’s upper limit were assigned the upper limit. Receiver operating characteristic (ROC) analysis was performed to assess the discriminative ability of day-of-injury hsCRP or GFAP for predicting PTSD at 6 months postinjury for all mTBI cases. The area under the ROC curve (AUC) was calculated with its 95% confidence interval.

Multivariable logistic regression models assessed whether day-of-injury levels of hsCRP or GFAP were independent predictors of PTSD adjusting for known risk factors based on our prior models using demographics, injury characteristics and medical history [5]. The models also adjusted for sampling time (as 0–8, 9–16, or 17–24 h post-injury). Missing values in the baseline covariates were imputed using multiple imputation methods; [25] no outcome data were imputed [26]. As seen in Table 1, “PTA status” had the largest amount of missingness (8.7%) because unknown was treated as missing in the models; CT status had ~2% missing, and other baseline covariates had <1% missing. Pooled results from multiple imputed datasets were reported. Statistical significance was set as a *p*-value <0.05. Statistical analyses were conducted in R, version 4.1.2 (R Core Team, 2013).

**Table 1:** Demographic and clinical characteristics of the study cohort.

	Had PTSD outcome at 6m post-injury		Total	*p*-value
	No	Yes		
**Age** Median (IQR)	38 (26, 55)	38 (25, 55)	38 (26, 55)	0.396
**Years of Education** Median (IRQ)	12 (11, 14)	13 (12, 16)	13 (12, 16)	<0.001
**Patient Type**				
ED Discharge	114 (23.65%)	339 (29.66%)	453 (27.88%)	<0.001
Hospital admit no ICU	186 (38.59%)	488 (42.69%)	674 (41.48%)	
Hospital admit with ICU	182 (37.76%)	316 (27.65%)	498 (30.65%)	
Total	482 (100%)	1143 (100%)	1625 (100%)	
**Gender**				0.002
Male	351 (72.82%)	740 (64.74%)	1091 (67.14%)	
Female	131 (27.18%)	403 (35.26%)	534 (32.86%)	
Total	482 (100%)	1143 (100%)	1625 (100%)	
**Race**				0.117
White	376 (80.17%)	865 (75.94%)	1241 (77.18%)	
Black	72 (15.35%)	198 (17.38%)	270 (16.79%)	
Other	21 (4.48%)	76 (6.67%)	97 (6.03%)	
Total	469 (100%)	1139 (99.99%)	1608 (100%)	
**Hispanic**				<0.001
Non-Hispanic	343 (72.82%)	945 (83.04%)	1288 (80.05%)	
Hispanic	128 (27.18%)	193 (16.96%)	321 (19.95%)	
Total	471 (100%)	1138 (100%)	1609 (100%)	
**Injury Cause**				0.551
Road traffic incident	260 (54.05%)	645 (56.63%)	905 (55.86%)	
Incidental fall	141 (29.31%)	315 (27.66%)	456 (28.15%)	
Violence/assault	37 (7.69%)	70 (6.15%)	107 (6.6%)	
Other	43 (8.94%)	109 (9.57%)	152 (9.38%)	
Total	481 (99.99%)	1139 (100.01%)	1620 (100%)	
**Prior TBI**				0.129
No	309 (71.36%)	763 (67.34%)	1072 (68.45%)	
Yes	124 (28.64%)	370 (32.66%)	494 (31.55%)	
Total	433 (100%)	1133 (100%)	1566 (100%)	
**Psychiatric History**				0.121
No	383 (79.63%)	868 (75.94%)	1251 (77.03%)	
Yes	98 (20.37%)	275 (24.06%)	373 (22.97%)	
Total	481 (100%)	1143 (100%)	1624 (100%)	
**GCS ED Arrival**				0.114
13	32 (6.64%)	49 (4.29%)	81 (4.98%)	
14	83 (17.22%)	220 (19.25%)	303 (18.65%)	
15	367 (76.14%)	874 (76.47%)	1241 (76.37%)	
Total	482 (100%)	1143 (100.01%)	1625 (100%)	
**LOC**				0.964
No	62 (12.89%)	142 (12.47%)	204 (12.59%)	
Yes	396 (82.33%)	941 (82.62%)	1337 (82.53%)	
Unknown	23 (4.78%)	56 (4.92%)	79 (4.88%)	
Total	481 (100%)	1139 (100.01%)	1620 (100%)	
**Posttraumatic Amnesia (PTA)**				0.393
No	93 (19.33%)	212 (18.61%)	305 (18.83%)	
Yes	357 (74.22%)	831 (72.96%)	1188 (73.33%)	
Unknown	31 (6.44%)	96 (8.43%)	127 (7.84%)	
Total	481 (99.99%)	1139 (100%)	1620 (100%)	
**CT Intracranial Injury**				0.12
CT−	277 (60.61%)	725 (64.79%)	1002 (63.58%)	
CT+	180 (39.39%)	394 (35.21%)	574 (36.42%)	
Total	457 (100%)	1119 (100%)	1576 (100%)	
**Sampling Time (hours)**				0.351
0–8h	100 (20.75%)	274 (23.97%)	374 (23.02%)	
9–16h	148 (30.71%)	341 (29.83%)	489 (30.09%)	
17–24h	234 (48.55%)	528 (46.19%)	762 (46.89%)	
Total	482 (100.01%)	1143 (99.99%)	1625 (100%)	

## Results

A total of 1625 TRACK-TBI participants with day-of-injury GFAP and hsCRP measures and ED admission GCS 13–15 were available (see Supplementary Fig. 1: STROBE Diagram of study cohort). Of these, 1143 completed the PCL-5 assessment at 6-months post-injury (see Supplementary Fig. 2), and 227 (19.9%) had probable PTSD (PCL-5 ≤ 33) at 6-months post-injury. Detailed demographic and clinical characteristics of the study population, shown as a comparison of participants for whom this outcome measure was or was not available, are shown in Table 1, with the latter group more likely to be Hispanic, male, less educated, and to have been admitted to the ICU. The median age of the 1,143 patients included in these analyses was 38 (IQR: 25–55) years. The sample was predominantly male (64.7%) and 56.6% were injured in road traffic accidents. The median time between injury and blood draw was 15.1 (IQR: 8.5–20.0) h.

### Baseline factors associated with GFAP and hsCRP

**Fig. 1:**
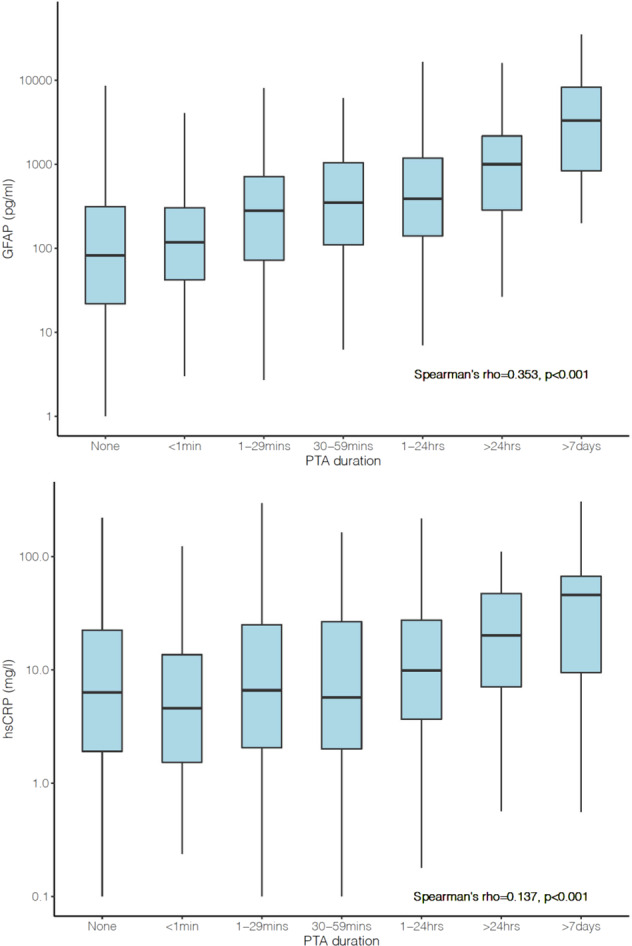
Relationship of Blood Biomarker Concentrations to Duration of Posttraumatic Amnesia. **a** (top): Day-of-injury plasma GFAP concentrations and duration of posttraumatic amnesia (PTA). **b** (bottom): Day-of-injury serum hsCRP concentrations and duration of posttraumatic amnesia (PTA).

There was a dose-dependent relationship (Spearman’s rho = 0.353, *p* < 0.001) between day-of-injury GFAP levels and duration of reported posttraumatic amnesia (PTA); those with higher GFAP levels had longer reported PTA (Fig. 1a). A relationship was also seen between day-of-injury hsCRP and PTA, albeit with hsCRP levels being elevated only among participants with PTA extending beyond 24 h (who would not meet ACRM criteria for mTBI) (Fig. 1b). Multivariable models assessing the association between baseline factors and (log)GFAP and (log)hsCRP are shown in Table 2a and Table 2b, respectively.

**Table 2:** Multivariable models.

	Coefficient (95% CI)	Wald's Chi-square	*p*-value
(a) Multivariable model assessing the association between baseline factors and day-of-injury (log)GFAP levels.			
**Age**	0.007 (0.002, 0.012)	8.61	0.003
**Sex** Female vs Male	−0.288 (−0.464, −0.113)	10.35	0.001
**Race**		3.07	0.215
Black vs White	−0.178 (−0.404, 0.049)		
Other vs White	0.103 (−0.232, 0.439)		
**Hispanic** Yes vs No	−0.092 (−0.308, 0.124)	0.7	0.404
**Injury cause** Violence vs Incident/other	−0.391 (−0.719, −0.064)	5.49	0.019
**Psychiatric history** Yes vs No	−0.216 (−0.408, −0.024)	4.85	0.028
**Prior TBI** Yes vs No	−0.283 (−0.458, −0.109)	10.1	0.001
**CT** + vs −	1.610 (1.431, 1.789)	309.7	<0.001
**PTA** Yes vs No	0.900 (0.704, 1.097)	80.5	<0.001
**Sampling Time**		32.45	<0.001
9–16h vs 0–8h	0.648 (0.424, 0.871)		
17–24h vs 0–8h	0.422 (0.214, 0.630)		

(b) Multivariable model assessing the association between baseline factor and day-of-injury (log)hsCRP levels.			
**Age**	0.009 (0.005, 0.013)	18.86	<0.001
**Sex** Female vs Male	−0.098 (−0.251, 0.055)	1.59	0.208
**Race**		4.01	0.134
Black vs White	0.081 (−0.116, 0.278)		
Other vs White	−0.252 (−0.544, 0.039)		
**Hispanic** Yes vs No	0.495 (0.307, 0.682)	26.64	<0.001
**Injury cause** Violence vs Incident/other	−0.233 (−0.518, 0.052)	2.57	0.109
**Psychiatric history** Yes vs No	−0.184 (−0.351, -0.016)	4.63	0.031
**Prior TBI** Yes vs No	−0.107 (−0.259, 0.045)	1.91	0.167
**CT** + vs −	0.321 (0.164, 0.477)	16.2	<0.001
**PTA** Yes vs No	0.183 (0.012, 0.354)	4.39	0.036
**Sampling Time**		629	<0.001
9–16h vs 0–8h	1.384 (1.190, 1.579)		
17–24h vs 0–8h	2.307 (2.125, 2.488)		

### GFAP and hsCRP values and PTSD Outcomes

**Fig. 2:**
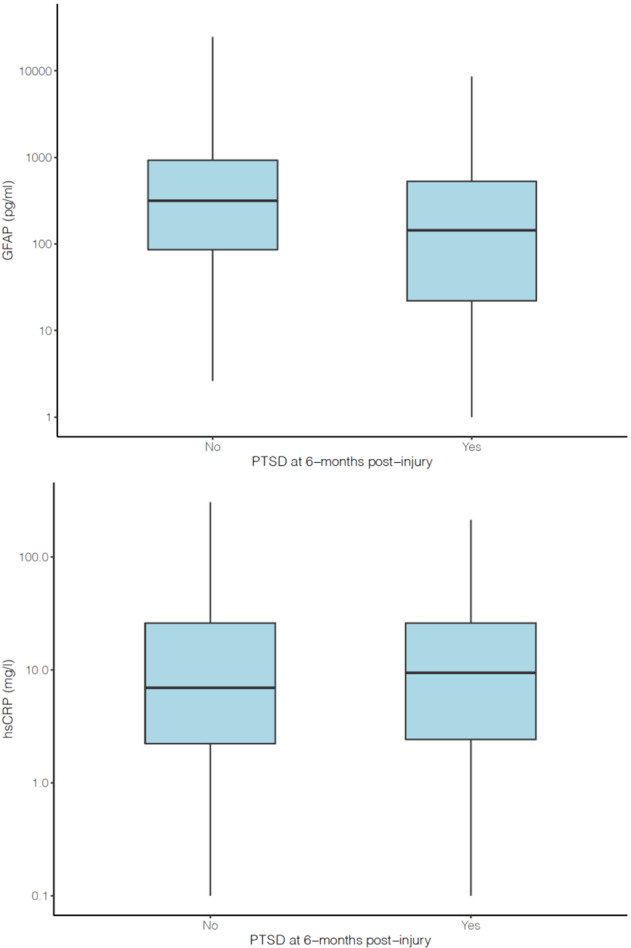
Day-of-injury blood biomarker concentrations among those without and with probable PTSD at 6-month outcome. **a** (top): Day-of-injury plasma GFAP concentrations among those without (“no”) and with (“yes”) probable PTSD at 6-month outcome. **b** (bottom): Day-of-injury serum hsCRP concentrations among those without (“no”) and with (“yes”) probable PTSD at 6-month outcome post-injury.

Day-of-injury GFAP values were significantly (*p* < 0.0001) lower in participants with (median: 142.9 [IQR: 21.0–529.3] pg/ml) versus those without (median: 314.0 [IQR: 84.6–924.3] pg/ml) probable PTSD at the 6-month assessment (Fig. 2a). The AUC for GFAP levels distinguishing between participants with and without probable PTSD at 6-months post-injury was 0.61 (95% CI: 0.57–0.66).

Day-of-injury hsCRP values did not significantly differ (*p* = 0.53) between participants with (median: 9.40 [IQR: 2.42–25.95] mg/L) and without (median: 6.93 [IQR: 2.21–25.96] mg/L) probable PTSD at the 6-month assessment (Fig. 2b). The AUC for hsCRP levels distinguishing between participants with and without probable PTSD at 6-months post-injury was no better than chance: 0.51 (95% CI: 0.47–0.56).

A multivariable logistic regression model including multiple known risk factors for PTSD (including age, sex, race, injury cause [violent vs. other], history of mental illness, CT scan results [positive vs. negative], history of prior TBI, and time of blood sampling) after TBI failed to show significant associations of (log)hsCRP with 6-month probable PTSD (adjusted OR = 1.11, 95% CI 0.98–1.25, *p* = 0.11), but continued to show significant associations of (log)GFAP with 6-month probable PTSD (adjusted OR = 0.85, 95% CI 0.77–0.95 per log unit increase; *p* = 0.003) (Table 3).

**Table 3:** Multivariable logistic regression model for predicting probable PTSD 6 Months post-injury (*N* = 1143).

	OR (95% CI)	*p*-value
**Age**	0.995 (0.986, 1.006)	0.322
**Sex** Female vs Male	1.425 (1.019, 1.994)	0.039
**Race** Black vs non-Black	2.816 (1.935, 4.098)	<0.001
**Injury cause** Violence vs Incident/other	2.656 (1.489, 4.736)	<0.001
**Psychiatric history** Yes vs No	2.408 (1.702, 3.406)	<0.001
**Prior TBI** Yes vs No	1.586 (1.138, 2.211)	0.007
**CT** + vs −	0.837 (0.555, 1.262)	0.395
**Sampling Time**		
9–16h vs 0–8h	1.347 (0.826, 2.196)	0.232
17–24h vs 0–8h	1.800 (1.104, 2.934)	0.019
**GFAP** (in log scale)	0.851 (0.766, 0.946)	0.003
**hsCRP** (in log scale)	1.105 (0.977, 1.251)	0.113

AUC = 0.723, 95% CI: (0.682, 0.76); Nagelkerke’s pseudo-R-squared = 0.168; likelihood ratio test comparing this model to the model without biomarkers and sampling time showed *p*-value < 0.001.

In a sensitivity analysis using the same predictors but excluding subjects with PTA > 24 h (who would not meet ACRM criteria for mTBI), we continued to see a significant association between higher (log)GFAP concentration and lower odds of 6-month probable PTSD (see Supplementary Table).

In a second sensitivity analysis using the same predictors in a multivariable linear regression with PCL-5 as a continuous outcome measure of PTSD symptoms, higher (log)GFAP was significantly associated with lower 6-month PCL-5 score (Table 4).

**Table 4:** Multivariable linear regression model for predicting PCL-5 Score 6 Months post-injury.

	Coefficient (95% CI)	*p*-value
**Age**	−0.067 (−0.125, −0.01)	0.021
**Sex** Female vs Male	3.953 (1.868, 6.039)	<0.001
**Race** Black vs non-Black	8.636 (6.018, 11.253)	<0.001
**Injury cause** Violence vs Incident/other	9.525 (5.389, 13.661)	<0.001
**Psychiatric history** Yes vs No	7.163 (4.844, 9.482)	<0.001
**Prior TBI** Yes vs No	3.523 (1.418, 5.628)	0.001
**CT** + vs −	0.215 (−2.189, 2.619)	0.861
**Sampling Time**		
9–16h vs 0–8h	1.765 (−1.108, 4.638)	0.229
17–24h vs 0–8h	3.355 (0.361, 6.348)	0.028
**GFAP** (in log scale)	−1.483 (−2.136, −0.831)	<0.001
**hsCRP** (in log scale)	0.600 (−0.159, 1.359)	0.122

## Discussion

Traumatic brain injury represents a significant health crisis in the United States and worldwide. The majority of TBIs are classified as mild (GCS 13–15) [9]. Although a majority of individuals that sustain a mild TBI will go on to recover completely, up to 20% will go on to suffer from psychiatric illness such as PTSD, particularly in the first 6 months post-injury [3, 5, 6]. With finite healthcare resources available, the ability to predict clinical outcomes to allocate resources toward individuals at the greatest risk of developing chronic post-TBI symptoms and disability would lead to both cost-savings and improvement in individual quality of life. This is particularly true for sequelae such as PTSD, for which proven clinical interventions exist. In fact, implementing clinical intervention as soon as possible following a traumatic event leads to decreased likelihood of developing PTSD [27].

Previous studies have indicated that there are certain demographic features such as sex, race, type of injury, and post-traumatic amnesia (PTA) that correlate with an increased risk of developing PTSD following TBI [5, 28]. However, many of these features, notably PTA, typically rely on subjective self-report. Recently, the blood-based biomarker, GFAP (a marker of astrocytic response to injury), has been identified as having clinical utility in evaluation of mTBI [16], a diagnosis which historically has also relied upon subjective symptom reporting and clinical judgement. It is therefore plausible that blood-based biomarkers could similarly be used to predict post-TBI mental health outcomes such as PTSD.

This study hypothesized that plasma levels of GFAP would correlate with PTA, a surrogate marker of injury severity [29], and with the development of PTSD. Additionally, because previous studies found a correlation between hsCRP, a non-specific peripheral marker of inflammation, and the development of PTSD [18], we hypothesized that serum hsCRP would also be associated with an increased risk for the development of PTSD, but to a lesser extent than the brain-specific marker GFAP. Consistent with our hypothesis, our data indicate that GFAP levels (which were on average substantially higher than those seen in uninjured healthy comparison subjects and in orthopedic trauma comparison subjects without TBI [30]) are associated in a dose-response fashion with duration of PTA (Fig. 1a). These data are in line with previous studies which demonstrated that both GFAP [12, 16] and duration of PTA are positively correlated with injury severity [29].

However, contrary to our hypothesis – which assumed that PTSD and TBI cause dysfunction in overlapping brain structures [31, 32] – GFAP levels were *inversely* correlated with the development of PTSD at 6 months post-injury (Table 3). Furthermore, hsCRP (Table 3), a non-specific marker of systemic inflammation, was not significantly associated with PTSD, indicating a brain-specific process. It is therefore possible that more extensive glial injury, reflected in higher levels of GFAP (and longer duration of PTA), interferes with encoding and/or consolidation of memories of the event, protecting against PTSD. In fact, previous research has indicated that shorter duration of PTA and memory of the event are predictive of PTSD [33]. Additionally, the hippocampus and its associated circuitry – structures vital to memory processes – are known to be particularly vulnerable to traumatic brain injury [34]. It will be of interest in future work to determine whether plasma GFAP levels reflect injury to specific brain structures or are a more global indicator of brain injury, and the extent to which interruption of critical neurocognitive processes impacts the development of PTSD in this context [35, 36].

GFAP levels were also significantly associated with numerous baseline factors (Table 2a). Unsurprisingly, higher levels of GFAP were seen in participants with abnormal CT scans or PTA, factors indicative of more severe injury. Lower levels of GFAP were seen in participants who were female, had an injury associated with violence, had a previous psychiatric diagnosis, or a history of TBI. It is possible that in this cohort, individuals with these baseline demographics sustained milder brain injuries. This is similar to previous studies in which less severe injury was associated with female sex and history of TBI [19], whereas more severe TBI occurred in high-impact injury mechanisms such as motor vehicle collisions and falls.

Our multivariable model included as covariates sociodemographic and patient historical characteristics which have previously been shown to be associated with PTSD following head injury [5], indicating that plasma GFAP concentration is an independent (negative) predictor of PTSD diagnosis. Although this is a promising finding, it should be noted that although an AUC of 0.72 (seen in the multivariable model) is higher than chance, it does not meet the level required for biomarker utilization in the clinical setting (typically > 0.75) [37]. It is likely that a panel of blood biomarkers (which might well in future include genetic markers) [38] in conjunction with structural [8] or functional [39] brain imaging characteristics, possibly in conjunction with other data-driven variables derived from machine learning approaches [40], will offer better predictive validity than single biomarkers such as GFAP alone.

### Strengths and limitations

Strengths of this study include its multi-center, longitudinal, prospective design, the large number of participants, use of multivariable statistical analysis, and measurement of plasma GFAP, which is already an FDA-approved biomarker to aid in the diagnosis of TBI by ruling out the need for a head CT scan. However, this study also has limitations. It was limited to adults and adolescents age 17 and older presenting to level 1 trauma centers who required a head CT scan, and had 6-month follow-up assessments. Accordingly, the findings may not be generalizable to individuals seen in community hospitals, military personnel, those without a clinical need for a CT scan, pediatric patients, those who sustained TBI but did not seek medical care, and individuals with characteristics or symptoms that prevented them from completing follow-up assessments. This study also relied on self-report of prior TBI, prior history of psychiatric illness, and PTA, which could lead to recall and reporting biases.

The gold standard for PTSD diagnosis remains a clinical interview that addresses DSM-5 criteria, but this study utilized the self-report PCL-5. Although the PCL-5 is a standardized assessment with good validity for making provisional PTSD diagnoses [21], the majority of questions do not pertain to memories of the event, and may identify individuals as potentially having PTSD when their symptoms are better attributed to mood or anxiety disorders.

## Conclusions

This study showed that day-of-injury GFAP plasma level, an objective biomarker cleared by FDA to assist in the diagnosis of TBI, was correlated with duration of PTA, indicating that duration of PTA is associated with more severe pathophysiological damage. Additionally, it is the first study to show that plasma GFAP levels were inversely associated with the development of PTSD at 6 months post-injury, suggesting that increased glial activation in response to injury may be *protective* against the development of PTSD. Astrocytes have a role in both amygdala plasticity and memory consolidation [41]. While our data suggest an inverse association between glial reaction to injury and the development of PTSD, other studies show that activated astrocytes are protective against PTSD [42]. Astrocytes are known to be complex, and have heterogenous responses dependent on injury mechanism and severity, including both adaptive and maladaptive properties [41]. Future research should be directed to investigating the role glia have in the development of PTSD in the context of TBI.

Importantly, addition of day-of-injury plasma GFAP improved the performance of previous PTSD prediction models which were based on participant demographics and injury characteristics. The current prognostic accuracy of GFAP does not meet the standards for clinical implementation to predict PTSD, but as its use as a blood-based biomarker for the extent of brain injury becomes more commonplace, clinicians should be aware that a low GFAP level does not indicate the absence of health risk to the patient. In fact, risk to mental health may be greatest in those with low (“normal”) GFAP levels. Future efforts should focus on using a panel of GFAP and other blood and possibly genetic biomarkers, combined with imaging modalities, to improve prediction of the development of PTSD and related mental disorders following mTBI.

## Supplementary information

Supplemental Material
