# Correction to: Association of day-of-injury plasma glial fibrillary acidic protein concentration and six-month posttraumatic stress disorder in patients with mild traumatic brain injury

**DOI:** 10.1038/s41386-022-01466-3

**Published:** 2022-09-30

**Authors:** Jacqueline R. Kulbe, Sonia Jain, Lindsay D. Nelson, Frederick K. Korley, Pratik Mukherjee, Xiaoying Sun, David O. Okonkwo, Joseph T. Giacino, Mary J. Vassar, Claudia S. Robertson, Michael A. McCrea, Kevin K. W. Wang, Nancy Temkin, Christine L. Mac Donald, Sabrina R. Taylor, Adam R. Ferguson, Amy J. Markowitz, Ramon Diaz-Arrastia, Geoffrey T. Manley, Murray B. Stein, TRACK-TBI Investigators, Neeraj Badjatia, Ann-Christine Duhaime, V. Ramana Feeser, C. Dirk Keene, Christopher Madden, Randall Merchant, Ava Puccio, David Schnyer, Sabrina R. Taylor, Alex Valadka, John K. Yue, Esther L. Yuh, Ross Zafonte

**Affiliations:** 1 Department of Psychiatry, University of California, San Diego, La Jolla, CA USA; 2 Biostatistics Research Center, Herbert Wertheim School of Public Health and Human Longevity Science, University of California, San Diego, La Jolla, CA USA; 3 Departments of Neurosurgery and Neurology, Medical College of Wisconsin, Milwaukee, WI USA; 4 Department of Emergency Medicine, University of Michigan, Ann Arbor, MI USA; 5 Department of Radiology & Biomedical Imaging, UCSF, San Francisco, CA USA; 6 Department of Bioengineering & Therapeutic Sciences, UCSF, San Francisco, CA USA; 7 Department of Neurological Surgery, University of Pittsburgh Medical Center, Pittsburgh, PA USA; 8 Department of Physical Medicine and Rehabilitation, Harvard Medical School, Boston, MA USA; 9 Spaulding Rehabilitation Hospital, Charlestown, MA USA; 10 Brain and Spinal Cord Injury Center, Zuckerberg San Francisco General Hospital and Trauma Center, San Francisco, CA USA; 11 Department of Neurological Surgery, UCSF, San Francisco, CA USA; 12 Department of Neurosurgery, Baylor College of Medicine, Houston, TX USA; 13 Department of Emergency Medicine, University of Florida, Gainesville, FL USA; 14 Department of Neurological Surgery, University of Washington, Seattle, WA USA; 15 Department of Neurology, University of Pennsylvania, Philadelphia, PA USA; 16 School of Public Health, University of California, San Diego, La Jolla, CA USA; 17 VA San Diego Healthcare System, San Diego, CA USA; 18 University of Maryland, College Park, MD USA; 19 MassGeneral Hospital for Children, Boston, MA USA; 20 Virginia Commonwealth University, Richmond, VA USA; 21 University of Washington, Seattle, WA USA; 22 UT Southwestern, Dallas, TX USA; 23 UT Austin, Austin, TX USA; 24 University of California, San Francisco, CA USA

**Keywords:** Diagnostic markers, Trauma

Correction to: *Neuropsychopharmacology* 10.1038/s41386-022-01359-5, published online 18 June 2022

In this article the GFAP was measured in plasma, not serum. GFAP can be measured in either serum or plasma, but all samples in this study were measured in plasma. The article was corrected accordingly.

The title was corrected accordingly from “Association of day-of-injury serum glial fibrillary acidic protein concentration and six-month posttraumatic stress disorder in patients with mild traumatic brain injury” to “Association of day-of-injury plasma glial fibrillary acidic protein concentration and six-month posttraumatic stress disorder in patients with mild traumatic brain injury”.

None of the data change as a result of this clarification.

None of the scientific conclusions of the paper are affected.

The original article has been corrected.

