# Supplementary material for: Association of day-of-injury plasma glial fibrillary acidic protein concentration and six-month posttraumatic stress disorder in patients with mild traumatic brain injury
Source: Neuropsychopharmacology. 2022 Jun 18;47(13):2300–8. doi: 10.1038/s41386-022-01359-5 (PMC9630517; doi:10.1038/s41386-022-01359-5)
Supplement: Supplementary file 1 — Supplemental Material [file 41386_2022_1359_MOESM1_ESM.pdf]

Supplemental Figure 1: STROBE DIAGRAM

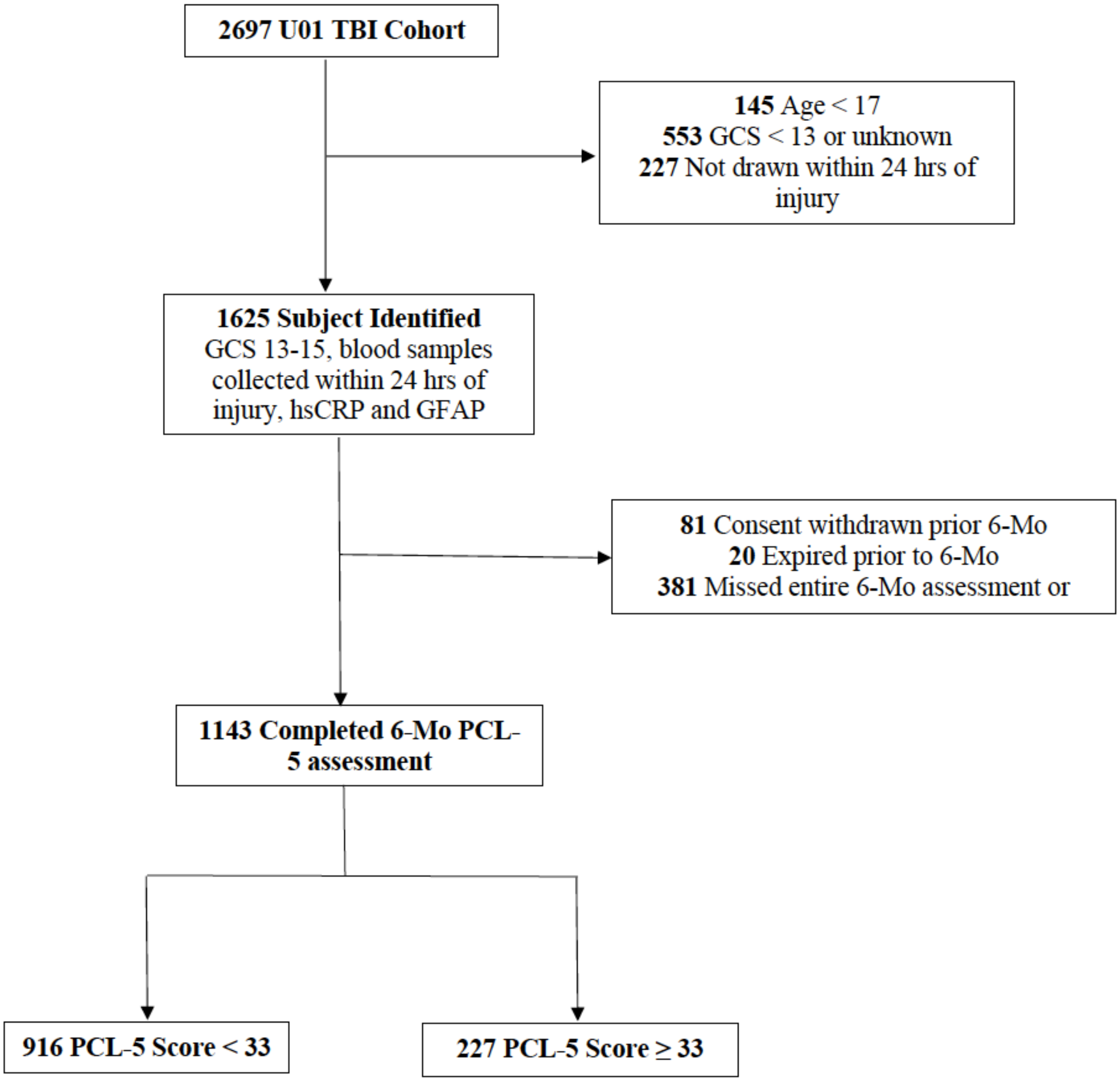

Supplemental Figure 2: Histogram of PCL-5 Total at 6m post-injury

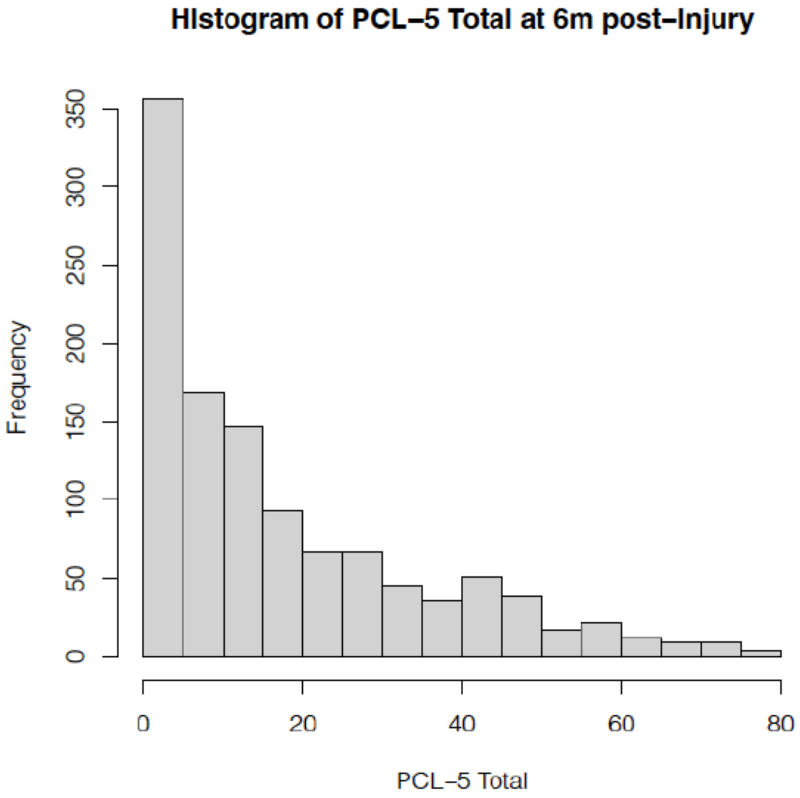

Supplemental Table: Sensitivity Model excluding subjects with posttraumatic amnesia > 24 hours (n=44 excluded; n=1099 included)

	OR	OR.l	OR.u	p.value
Age	0.995	0.985	1.005	0.285
Sex_Female	1.341	0.952	1.89	0.094
Race_Black	2.904	1.982	4.255	<0.001
InjCause_Violence	2.531	1.394	4.594	0.002
Psychiatric History	2.573	1.805	3.67	<0.001
PriorTBI_yes	1.629	1.162	2.284	0.005
CT_abnormal	0.848	0.554	1.298	0.447
SamplingTime9-16h	1.38	0.841	2.265	0.203
SamplingTime17-24h	1.856	1.129	3.051	0.015
logGFAP	0.861	0.774	0.959	0.007
loghsCRP	1.091	0.962	1.237	0.175
